# The Usefulness of Vanin-1 and Periostin as Markers of an Active Autoimmune Process or Renal Fibrosis in Children with IgA Nephropathy and IgA Vasculitis with Nephritis—A Pilot Study

**DOI:** 10.3390/jcm11051265

**Published:** 2022-02-25

**Authors:** Małgorzata Mizerska-Wasiak, Emilia Płatos, Karolina Cichoń-Kawa, Urszula Demkow, Małgorzata Pańczyk-Tomaszewska

**Affiliations:** 1 Department of Pediatrics and Nephrology, Medical University of Warsaw, 02-091 Warsaw, Poland; karolina.cichon@yahoo.com (K.C.-K.); mpanczyk1@wum.edu.pl (M.P.-T.); 2 Science Students’ Association at the Department of Pediatrics and Nephrology, Medical University of Warsaw, 02-091 Warsaw, Poland; emiliaplatos@gmail.com; 3 Department of Laboratory Diagnostics and Clinical Immunology of Developmental Age, Medical University of Warsaw, 02-091 Warsaw, Poland; urszula.demkow@wum.edu.pl

**Keywords:** IgA nephropathy, IgA vasculitis with nephritis, vanin-1, periostin, biomarker, children

## Abstract

This study aimed to evaluate the usefulness of vanin-1 and periostin in urine as markers of the autoimmune process in kidneys and renal fibrosis in IgA nephropathy (IgAN) and IgA vasculitis with nephritis (IgAVN). From a group of 194 patients from the Department of Pediatrics and Nephrology, who were included in the Polish Pediatric Registry of IgAN and IgAVN, we qualified 51 patients (20 with IgAN and 31 with IgAVN) between the ages of 3 and 17, diagnosed based on kidney biopsy, for inclusion in the study. All of the patients received glucocorticosteroids, immunosuppressive drugs, or renoprotective therapy. The control group consisted of 18 healthy individuals. The concentration of vanin was significantly higher in the IgAN and IgAVN groups than in the control group. The concentration of vanin/creatinine correlates positively with the level of IgA and negatively with the serum level of C3 at the end of the observation. Urinary vanin-1 concentration may be useful as a marker of the active autoimmune process in IgAN and IgAVN in children, but the study needs confirmation on a larger group of children, along with evaluation of the dynamics of this marker. Urinary periostin is not a good marker for children with IgAN and IgAVN, especially in stage 1 and 2 CKD.

## 1. Introduction

IgA nephropathy (IgAN) is the most frequently diagnosed type of primary glomerulonephritis worldwide [1,2,3]. Its estimated incidence is 2.5/100,000/year; however, it varies across geographical locations. In Asia, the incidence rate is statistically higher than in Europe or the Americas [4,5]. Scientific research demonstrated an up to 8-fold higher increased frequency of occurrence of IgAN among Japanese children and teenagers than among American children [6,7].

IgA vasculitis (IgAV), previously known as Henoch–Schönlein purpura (HSP), is the most common cause of systemic childhood vasculitis [8,9,10]. Although its etiology remains vague, the strong predisposition among individuals with the HLA-DRB1*01 allele was revealed [8,9]. IgAV affects small vessels of the skin, gastrointestinal tract, and joints. Nonetheless, immunoglobulin A (IgA) vasculitis with nephritis (IgAVN) manifests in about one-third of IgAV patients [11].

Hallmarks of IgAN, a disease described by Burger and Hinglaise in 1968, are IgA-dominant deposits with co-dominant IgG and IgM deposits in mesangial glomeruli. Although complement C3 is almost always detected, C1q remains mostly absent [12,13]. The pathogenesis model is described as “multi-hit” and composed of a few stages: elevated galactose deficient IgA1 levels and the generation of anti-glycan antibodies, resulting in the formation of immune complexes out of themselves. Accumulation of those deposits leads to the activation of the alternative complement pathway and mesangial proliferation with inflammatory and fibrotic effects [12,14,15]. Acute kidney injury (AKI) occurs in up to 10% of IgAN patients and belongs to the risk factors for renal disease [16,17]. IgAN may lead to end-stage renal failure (ESRF) in up to 60% of cases [18].

The Oxford MEST-C Score facilitates the assessment of renal biopsy samples. It defines risk factors of kidney insufficiency and can be used in IgAVN patient samples [19,20].

IgAVN, despite different disease courses, has some common features with IgAN, which apply to a similar pathomechanism, linked to IgA complexes [21,22,23]. Although IgAN and IgAV are considered to be similar, they can be distinguished from each other with clinical manifestation or peak age ranges of diagnosis [24]. More recent evidence (Ozen et al., 2019) proposes SHARE recommendations for IgAV/IgAVN as a practical source of information. These recommendations underline the importance of renal and skin biopsy, eGFR, urinalysis, and ultrasound [25].

Vanin-1 has a crucial role in the recycling of pantothenic acid and is a precursor of CoA in the long term [26]. Since its discovery in 1945, coenzyme A has been known as a cofactor of lipid metabolic processes and energy production [27]. That explains its remarkable expression in tissues with CoA turnover, e.g., kidney, liver, or intestine [28]. Vnn1, which codes vanin-1, is involved in numerous metabolic pathways. For instance, in mice, it upregulates peroxisome proliferator-activated receptor alpha (PPAR-α)—liver’s fasting regulator [29]. Proximal tubuli of the kidney are one of the places of vanin-1 expression [26]. Vanin-1 is a novel tissue sensor for oxidative stress and its level increases in AKI before the emergence of classic markers, e.g., N-acetyl-β-d-glucosaminidase (NAG), creatinine, or blood urea nitrogen (BUN) [30]. Vanin helps detect AKI, particularly kidney injuries triggered by nephrotoxic drugs and intoxication [31,32].

Novel studies have suggested its relevance to the prediction and evaluation of obstructive nephropathy (hydronephrosis) [32]. In addition, pantetheine, the substrate of vanin-1, may improve the vasculopathy observed in several inflammatory conditions. Enhanced levels of vanin-1 can be related to both positive and negative autoimmune disease prognoses [33,34].

Periostin is a protein expressed in multiple tissues during embryonal development; however, it is detected in collagen-rich tissues such as bone, dental tissues, and heart valves as well as in injured areas, e.g., kidneys [35,36,37,38]. It integrates an extracellular matrix (ECM) in physiological conditions; nonetheless, overexpression may lead to organ fibrosis [36,37]. AKI progression follows periostin expression and leads to renal fibrosis in response to hypoxia or ischemia [39]. That suggests its potentiality to become a novel CKD biomarker [35,37,40,41]. Patients with glomerulopathies might have increased periostin expression in the mesangium, tubular interstitium, and even areas of fibrosis according to biopsy findings. Interestingly, the external addition of TGF-β1 also leads to extreme periostin production [42]. De novo overexpression of periostin was found in several renal diseases such as lupus nephritis, diabetic nephropathy, IgA nephropathy, and focal-segmental glomerulosclerosis [42,43,44,45]. Moreover, complex periostin–human IgA1 in a ratio 1:1 might play a role in the pathomechanism of allergic diseases [46]. Although the trigger factors and mechanisms of periostin overexpression should still be elucidated, the interaction of periostin with ECM was extensively clarified [38].

This study aimed to evaluate the significance of vanin-1 and periostin in urine for the autoimmune inflammatory process in kidneys and renal fibrosis in IgAN and IgAVN.

## 2. Materials and Methods

We performed a prospective, cross-sectional study.

From the group of 194 patients hospitalized in the Department of Pediatrics and Nephrology with diagnosed IgAN or IgAVN, who were included in the Polish Pediatric Registry of IgAN and IgAVN, we qualified 51 patients (20 with IgAN and 31 with IgAVN) between the ages of 3 and 17, diagnosed based on kidney biopsy, for inclusion in the study. The control group consisted of 18 healthy individuals after detailed physical examination without any symptoms of the diseases and any chronic illnesses in their past medical history. The patients included in the study fulfilled the following inclusion criteria: IgAN or IgAVN diagnosis based on renal biopsy with histological proof of predominant mesangial IgA immune deposits remained under the supervision of doctors in the clinic. All of the biopsy samples were screened by optical, immunofluorescence, and electron microscopy.

### 2.1. Clinical and Biological Features

The parameters assessed at the onset of the disease were age, weight, sex, protein in the 24 h urine collection, protein, and erythrocytes in the urinalysis, eGFR, creatinine, albumin, cholesterol, triglycerides, IgA, IgM, IgG, and complement components: C3 and C4. To diagnose the disease in all patients, we performed a histopathological examination of the material collected during the kidney biopsy. After treatment administration, we implemented follow-up measurements and tested the levels of vanin and periostin in the urine. 

For each patient, their age, weight, and sex were determined by a nurse when the patient was admitted to the ward. General urine test samples, containing approximately 20 mL of midstream urine, were collected in plastic containers. Moreover, 50 mL of urine samples taken during the 24 h urine collection was transferred to plastic containers. Furthermore, 4 mL whole blood samples were collected in lithium heparin tubes aimed at the determination of creatinine, cholesterol, triglyceride levels, and complement components: C3 and C4 levels. To assess the level of albumin and immunoglobulins IgA, IgM, and IgG, 5 mL of whole blood was collected in test tubes with a separating gel. The material was stored at room temperature, centrifuged for 5 min at 4000 rpm, transferred to new tubes, and sent afterward to the hospital diagnostic laboratory.

A Human Vanin-1 (urine) ELISA Kit and Human Periostin ELISA Kit were used to test vanin and periostin levels. Ten mL urine samples were collected from each patient. The sensitivity of the Vanin-1 ELISA Kit was 9.6 pmol/L (=500 pg/mL) and the vanin concentration was converted according to the formula 1 pmol/L = 52.07 pg/mL (MW: 52.07 kDa). The sensitivity of the Periostin ELISA Kit was 20 pmol/L (=1800 pg/mL) and the periostin concentration was converted according to the formula 1 pmol/L = 91 pg/mL (MW: 91 kDa).

### 2.2. Renal Biopsy

After the detection of unexplained hematuria and proteinuria, the patients were qualified for renal biopsy. The diagnostic material was obtained from a large-needle percutaneous biopsy performed under ultrasound guidance. Three bioptates were evaluated under the light/electron microscope or in the immunofluorescence tests. A total of 25–50 serial 2–5 µm thick sections were analyzed. Direct immunofluorescence was performed with fluorescein-conjugated antibodies (FITC). A clear green fluorescence was assumed as a positive reaction, rated between 0 and +4. Immunomorphological diagnostics included the performance of reactions with antibodies against IgG, IgA, IgM, C3, C1q, fibrinogen, albumin, lambda, and kappa light chains. Findings were categorized according to the Oxford MEST-C score (M—mesangial hypercellularity: M0 > 50%, M1 < 50%; E—endocapillary hypercellularity: 0—absent, 1—present; S—segmental sclerosis/adhesion: 0—absent, 1—present; T—tubular atrophy/interstitial fibrosis: T0 0–25%, T1 26–50%, T2 > 50%; C—crescents: C0 0%, C1 0–25%, C2 > 25%. The overall score is calculated as the sum of M, E, S, T, and C).

### 2.3. Treatment

The patients received glucocorticosteroids (prednisone, deflazacort), immunosuppressive drugs (azathioprine, cyclophosphamide, cyclosporin A), renoprotective therapy (angiotensin-converting enzyme inhibitor (ACEI)/angiotensin receptor blocker (ARB): enalapril, amlodipine), and/or omega-3 fatty acid. 

### 2.4. Monitoring and Follow-Up

Patients remain under the supervision of pediatric nephrologists at the Department of Pediatrics and Nephrology, Medical University of Warsaw. The development of their diseases is continuously monitored.

The study was approved by the Bioethics Committee at the Medical University of Warsaw (No. KB/147/2017).

**Figure 1:**
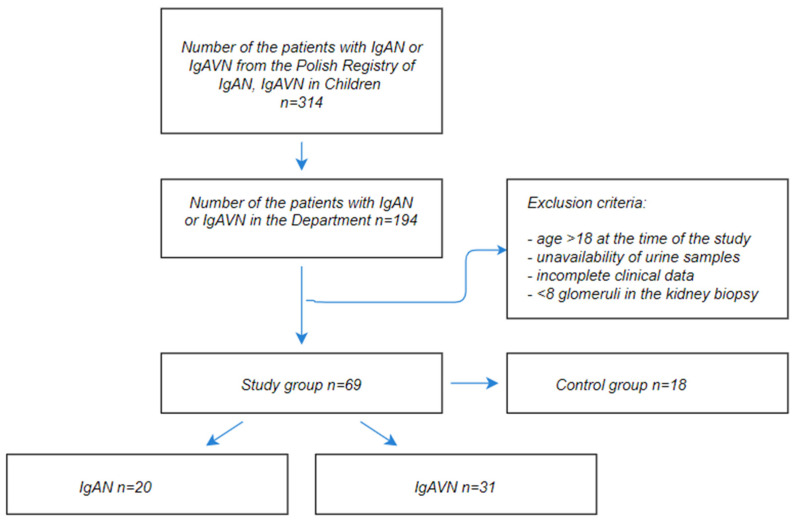
The flow diagram of the study.

The flow diagram of the study is shown in Figure 1.

Statistical analysis was performed with Statistica 13 (maintained by TIBCO Software, Inc., Palo Alto, CA, USA) using the Student’s *t*-test for normally distributed variables: age, height, weight, and their values on growth charts, glomerular filtration rate (GFR), albumin, cholesterol, triglycerides, IgG, IgM, C3, C4, and the sum of the MEST-C score. For non-normally-distributed variables: proteinuria mg/kg/day, erythrocyturia, urea, creatinine, IgA, time to biopsy, vanin (pmol/L, pg/mg creatinine), and periostin (pg/mg creatinine), we used the Mann–Whitney test. The Kruskal–Wallis test was used to compare the values of vanin and periostin in the groups of IgAN and IgAVN patients with the control group (three groups). To evaluate differences between the baseline and follow-up values, the Student’s *t*-test and the Wilcoxon test were used (for normally and non-normally distributed variables, respectively). Linear regression analysis and Spearman’s rank correlation were also performed. The Mann–Whitney test was used to compare the individual components of MEST-C in the IgAN and IgAVN groups. The generalized GRM linear regression method helped us to find qualitative (group, gender) and quantitative variables that correlate with vanin, periostin, vanin/cr, and periostin/cr. Using the step-wise method, statistically, significant variables correlated with the above-mentioned variables were found. 

Independently, the correlation between the variables was investigated using Spearman’s rank method.

## 3. Results

The mean age at the diagnosis of IgAN/IgAVN was 8.84 ± 3.88 years. At baseline, the mean proteinuria was 28.8 (0–742) mg/kg/d; erythrocyturia was 80 (6–250) HPF; GFR was 108.89 ± 30.62 mL/min; IgA was 250.95 (57.8–1070); C3 was 109.65 ± 28.96; and C4 was 23.93 ± 7.89 mg/dL. 

Renal biopsy was performed 1.0 (0–80.4) months after the onset of the disease. 

The duration of follow-up was 3.31 ± 2.78 years. At the end of this period, proteinuria was 0 (0–180) mg/kg/d, erythrocyturia was 0 (0–200) HPF; GFR was 112.48 ± 17.71 mL/min; IgA was 192.1 (48.1–439) mg/dL; C3 was 93.62 ± 14.78 mg/dL; and C4 was 18.11 ± 6.9 mg/dL.

The clinical characteristics of the study patients divided into two groups, IgAN (group 1) and IgAVN (group 2), are shown in Table 1.

**Figure 2:**
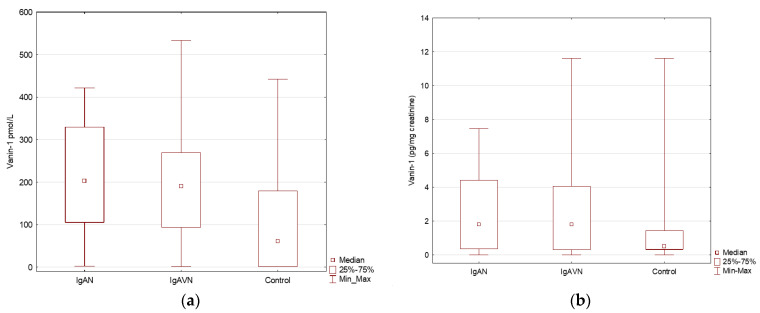
The concentration of vanin and vanin/creatinine in urine samples in children with IgAN, IgAVN, and in the control group. (**a**) The concentration of vanin in urine samples in IgAN, IgAVN and control group; (**b**) The concentration of vanin/creatinine in urine samples in IgAN, IgAVN and control group.

Subgroup analyses were also performed for the vanin and periostin in urine and further converted to creatinine, as shown in Table 2 and Figure 2.

**Figure 3:**
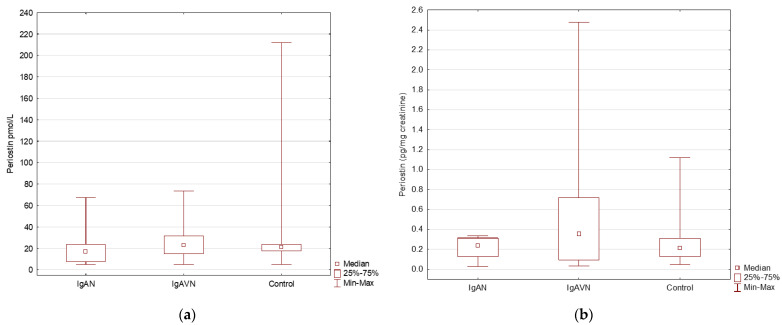
The concentration of periostin and periostin/creatinine in the urine samples in children with IgAN, IgAVN, and in the control group. (**a**) The concentration of periostin in urine samples in IgAN, IgAVN and control group; (**b**) The concentration of periostin/creatinine in urine samples in IgAN, IgAVN and control group.

The concentration of vanin was significantly higher in the IgAN and IgAVN groups than in the control group (*p* < 0.05). Furthermore, no significant differences were found in the concentration of vanin between the IgAN and IgAVN groups. In the study group, periostin concentration did not differ between the IgAN, IgAVN, and control groups (Figure 3).

**Figure 4:**
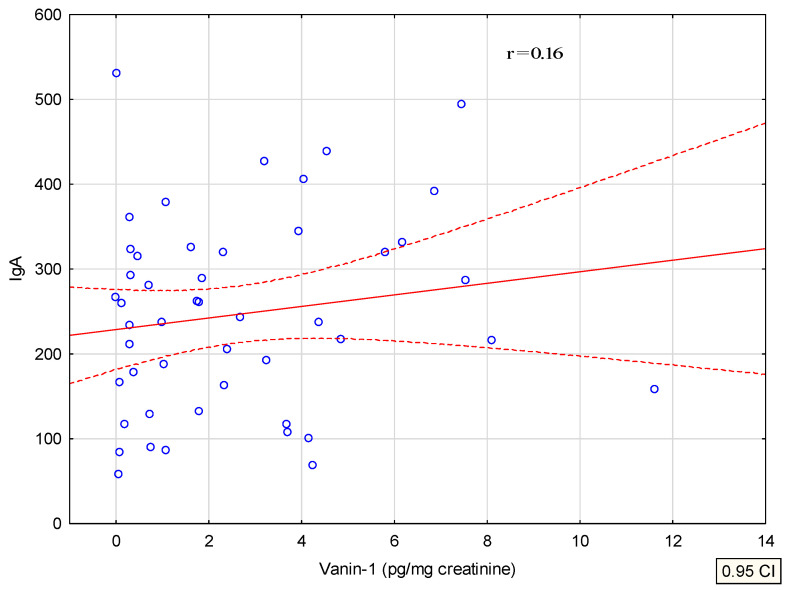
Correlation of vanin with serum IgA.

**Figure 5:**
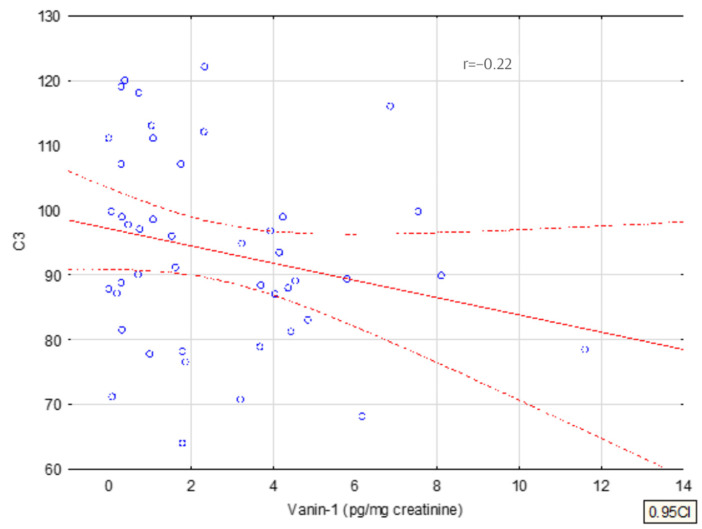
Correlation of urinary vanin with serum C3.

A multivariate analysis of the concentration of vanin and periostin in urine was performed and the significant correlations were presented on a graph of linear correlations, as shown in Figure 4 and Figure 5. The only statistically significant differences (*p* < 0.05) we observed were for the vanin-1 and vanin-1/creatinine concentration among patients with IgAVN and the whole study group (IgAVN and IgAN).

The concentration of vanin/cr correlates positively with the level of IgA and negatively with the serum level of C3 at the end of the observation. 

The regression analysis confirmed a positive correlation of periostin and vanin (in pmol/l and ng/mg creatinine) in the IgAVN group and the whole study group. 

In the multivariate GRM analysis (general regression model) of the dependence of vanin concentration, a weak negative correlation with C3 at the end of the observation and a positive correlation with the SDS of body weight was confirmed, as shown in Table 3.

GRM analysis did not confirm a correlation of periostin with the tested parameters. 

We analyzed the results of kidney biopsy in the Oxford classification, and the levels of vanin and periostin in urine depending on the MEST-C score.

In the IgAN and IgAVN groups, no significant differences were found in the concentration of vanin or periostin in urine in E1, S1, T1-2, or C1-2 compared to E0, S0, T0, and C0 (M1 occurred in all IgAN patients).

The treatment analysis of children in the study group confirmed that 61% of all patients received immunosuppressive therapy, including 71% in the IgAVN group and 45% in the IgAN group, and therefore it should not influence the better or worse treatment of some groups of the patients. 

## 4. Discussion

Vanin-1 had not been investigated among children nor adults with IgAN or IgAVN until now. Studies in the scope of periostin’s role in IgAN include only adults and relate mostly to serum periostin levels. Thus, this study is a novelty and it was planned as a pilot study on a small group of patients.

The subgroups within the study group differed significantly only in the age of diagnosis and the time from diagnosis to a kidney biopsy, which results from the natural course of these diseases and the usually faster development of IgAVN.

In our study, we demonstrated the significant growth of urinary vanin-1 levels in children with IgAN or IgAVN diagnosis in comparison to the control group. Hosohata et al. were some of the first researchers who observed increased levels of urinary vanin-1 24 h after exposure to toxic solvents, and thus suggested vanin-1 to be a predictive biomarker of AKI [40]. GFR reduction at the onset of the disease was confirmed in nine patients (five with IgAN, four with IgAVN), whereas at the end of follow-up GFR < 90 mL/min had only two pediatric patients with IgAVN (GFR of 86 and 80 mL/min). 

Although vanin-1 expression takes place in proximal tubuli, not in glomeruli, which would be remarkable for our findings, tubular atrophy is also recognized as one of the Oxford classifications (MEST-C). The MEST-C score enables stratification of the risks of IgAN nephropathy progression regarding, e.g., mesangial and endocapillary hypercellularity–proof of glomerular injury, or interstitial fibrosis/tubular atrophy–tubular compartment [20]. In our study, we did not observe significant differences between the levels of vanin in patients with T0 and T1-2.

The concentration of urinary vanin-1 correlates negatively with simultaneously measured serum C3 levels. However, in our study, we did not find the prognostic significance of the reduction of C3 in the serum of children with IgAN, which furthermore cannot confirm the prognostic significance of vanin [47].

The lack of significant differences, dependent on the Oxford-MEST-C score, might result from the time between kidney biopsy and vanin uptake and the influence of the implemented treatment on reversibility MEST-C classification elements.

There were no significant differences in the concentration of periostin in urine between the study group and the control group. Nevertheless, a relationship between periostin in the serum and the presence of fibrosis in the kidney tissue has been found in the studies performed so far. This relationship was shown in the study by Jia et al., especially for patients with severe CKD. Our group included children with CKD 1 and 2, and at the time of periostin uptake, only two patients presented CKD 2 [48].

Hwang et al. observed a positive correlation of periostin concentration in urine with interstitial fibrosis, interstitial inflammation, and glomerulosclerosis. Higher periostin levels in urine were linked to greater filtration failure in long-term observation, particularly with GFR < 60 mL/min. In our research, we have not confirmed this dependence, which might be associated with normal GFR in the long-term follow-up in 49 out of the 51 patients studied. Periostin levels were not tested during the renal biopsy, which may also play an important role [45].

Satirapoj et al. found that, in adults with diabetes mellitus type 2, urinary periostin correlates negatively with GFR and the severity of proteinuria; however, the investigated patients with an eGFR between 45.9 and 69.9 mL/min had a history of proteinuria. In our group, median proteinuria was 0, and mean GFR = 112.48 mL/min. It would be appropriate to investigate the level of microalbuminuria [44].

In Turczyn et al., in children with congenital obstructive nephropathy, a positive correlation of periostin with serum creatinine and cystatin C was found, with the presence of severe and moderate renal scars and borderline lesions in scintigraphy; in our study, periostin was measured after intensive treatment with positive effect for GFR and proteinuria [49].

### Limitations

One of the limitations of our study is the lack of measurement of vanin and periostin concentration in the urine at the onset of the disease (at the time of the kidney biopsy); however, this is a pilot study.

## 5. Conclusions

Urinary vanin-1 concentration may be a biomarker of active inflammation in IgAN and IgAVN in children, but the study needs confirmation on a larger group of children, along with evaluation of the dynamics of this marker.

Urinary periostin is not a good marker for children with IgAN and IgAVN, especially in stage 1 and 2 CKD, but the study needs confirmation on a larger group and in higher stages of the disease progression.

## Figures and Tables

**Table 1:** Clinical characteristics of the IgAN and IgAVN patient groups.

Parameter	IgAN (*n* = 20)	IgAVN (*n* = 31)	*p*
Age at the disease onset (years)	11 ± 4.1	7.5 ± 3.1	0.001
Height (cm)	150.2 ± 26.6	129.2 ± 20.2	0.002
Weight (kg)	45.8 ± 20.8	31.8 ± 14.6	0.007
SDS to weight	0.6 ± 1.4	0.3 ± 0.9	NS
GFR (mL/min/1.73 m^2^)	101.8 ± 30	113.5 ± 30.6	NS
Proteinuria at baseline (mg/kg/d)	19 (0–226)	34 (0–742)	NS
Erythrocyturia (RBC/HPF)	65.5 (0–250)	80 (0–250)	NS
Urea (mg/dL)	228 (18–43)	27 (15–159)	NS
Creatinine (mg/dL)	0.6 (0.3–1.3)	0.4 (0.3–2.1)	0.005
Albumin (g/dL)	3.7 ± 0.8	3.6 ± 0.7	NS
IgA (mg/dL)	302 (57.8–494)	213.4 (68.7–1070)	0.032
C3 (mg/dL)	106.6 ± 22.9	111.6 ± 32.5	NS
C4 (mg/dL)	24.8 ± 8	23.4 ± 7.9	NS
MEST-C score (sum)	2.2 ± 1.4	2.5 ± 1.2	NS
Time to biopsy (months)	3 (0–68.4)	1 (0–26)	0.03
Outcome (years)	2.9 ± 2.6	3.6 ± 2.9	NS
Age at FU (years)	14.1 ± 3.7	10.8 ± 3.6	0.004
Height at FU (cm)	154.4 ± 27.4	144.7 ± 18.6	NS
Weight at FU (kg)	53.6 ± 20.9	45.8 ± 19.8	NS
SDS to weight at FU	0 ± 1.5	0.7 ± 1.3	NS
GFR at FU (mL/min/1.73 m^2^)	111 ± 14.6	113.4 ± 19.6	NS
Proteinuria at FU (mg/kg/d)	0 (0–76)	0 (0–190)	NS
Erythrocyturia at FU (RBC/HPF)	6 (0–200)	0 (0–35)	0.002
Creatinine at FU (mg/dL)	0.6 ± 0.2	0.6 ± 0.1	NS
Albumin at FU (g/dL)	4.2 ± 0.6	4.4 ± 0.2	NS
IgA at FU (mg/dL)	253 (51–439)	173.2 (49.1–396)	0.033
C3 at FU (mg/dL)	91.3 ± 13.6	95 ± 15.5	NS
C4 at FU (mg/dL)	18 ± 7.1	18.2 ± 6.9	NS

BMI = Body mass index; GFR = glomerular filtration rate; FU = follow up; MEST-C score = the Oxford classification of IgA nephropathy; SDS = standard deviation score; NS = not significant.

**Table 2:** Vanin and periostin levels in the IgAV and IgAVN patient groups and the control group.

Parameter	Control Group (*n* = 18)	IgAN (*n* = 20)	IgAVN (*n* = 31)	*p*
Vanin (pmol/L)	61.1 (1–442.1)	203.4 (2.5–421.6)	190.4 (1.1–533)	0.016
Periostin (pmol/L)	21 (5–212.1)	16.7 (5–67.5)	22.8 (5–73.4)	NS
Vanin (pg/mg creatinine) *	0.5 (0.0–11.6)	1.8 (0.0–7.5)	1.8 (0–11.6)	NS
Perostin (pg/mg creatinine) *	0.2 (0.1–1.1)	0.2 (0.0–153.3)	0.4 (0.0–2.5)	NS

* pg/mg creatinine: We obtained the results in this parameter to adjust the results according to the gender of our patients and the different intensities of creatinine elimination; NS = not significant.

**Table 3:** General regression model analysis results for vanin (pg/mg creatinine).

	Subgroup	Vanin (pg/mg Creatinine) Parameter Correlation	−95% CI	+95% CI	*p*
SDS to weight at FU		0.6757	−0.0755	1.2760	0.0285
GFR at FU		0.0075	−0.0416	0.0565	0.7582
C3 at FU		−0.0561	−0.1079	0.0047	0.0711
Group	IgAN	0.1450	−0.7856	1.0757	0.7533
Sex	F	−0.1197	−0.9952	0.7559	0.7827

FFU = foFFUFU = follow up; CI = confidence interval; SDS = standard deviation score; GFR = glomerular filtration rate.

## Data Availability

The data analyzed in this study are available from the corresponding author upon reasonable request.
